# Anticoagulation in older people with atrial fibrillation moving to care homes: a data linkage study

**DOI:** 10.3399/BJGP.2022.0156

**Published:** 2022-12-30

**Authors:** Leona A Ritchie, Stephanie L Harrison, Peter E Penson, Ashley Akbari, Fatemeh Torabi, Joe Hollinghurst, Daniel Harris, Oluwakayode B Oke, Asangaedem Akpan, Julian P Halcox, Sarah E Rodgers, Gregory YH Lip, Deirdre A Lane

**Affiliations:** Liverpool Centre for Cardiovascular Science, University of Liverpool; Liverpool Heart & Chest Hospital; Department of Cardiovascular and Metabolic Medicine, Institute of Life Course and Medical Sciences, University of Liverpool, Liverpool, UK.; Liverpool Centre for Cardiovascular Science, University of Liverpool; Liverpool Heart & Chest Hospital; Department of Cardiovascular and Metabolic Medicine, Institute of Life Course and Medical Sciences, University of Liverpool, Liverpool, UK.; Liverpool Centre for Cardiovascular Science, University of Liverpool; Department of Cardiovascular and Metabolic Medicine, Institute of Life Course and Medical Sciences, University of Liverpool; School of Pharmacy and Biomolecular Sciences, Liverpool John Moores University, Liverpool, UK.; Population Data Science, Health Data Research UK and Population Data Science, Administrative Data Research Wales, Swansea University Medical School, Swansea University, Swansea, UK.; Population Data Science, Health Data Research UK, Swansea University Medical School, Swansea University, Swansea, UK.; Population Data Science, Health Data Research UK, Swansea University Medical School, Swansea University, Swansea, UK.; Population Data Science, Health Data Research UK, Swansea University Medical School, Swansea University, Swansea, UK.; Liverpool Centre for Cardiovascular Science, University of Liverpool; Liverpool Heart & Chest Hospital; Department of Cardiovascular and Metabolic Medicine, Institute of Life Course and Medical Sciences, University of Liverpool, Liverpool, UK.; Musculoskeletal and Ageing Science, Institute of Life Course & Medical Sciences, University of Liverpool; Liverpool University Hospitals NHS Foundation Trust, Liverpool, UK.; Population Data Science, Health Data Research UK, Swansea University Medical School, Swansea University, Swansea, UK.; Department of Public Health, Policy and Systems, Institute of Population Health, University of Liverpool, Liverpool, UK.; Price-Evans chair of cardiovascular medicine, consultant cardiologist, and distinguished professor;; Liverpool Centre for Cardiovascular Science, University of Liverpool; Liverpool Heart & Chest Hospital; Department of Cardiovascular and Metabolic Medicine, Institute of Life Course and Medical Sciences, University of Liverpool, Liverpool, UK; Aalborg Thrombosis Research Unit, Department of Clinical Medicine, Aalborg University, Aalborg, Denmark.

**Keywords:** anticoagulants, atrial fibrillation, long-term care, nursing homes, practice patterns, physicians’, primary health care

## Abstract

**Background:**

Treatment decisions about oral anticoagulants (OACs) for atrial fibrillation (AF) are complex in older care home residents.

**Aim:**

To explore factors associated with OAC prescription.

**Design and setting:**

Retrospective cohort study set in care homes in Wales, UK, listed in the Care Inspectorate Wales Registry 2017/18.

**Method:**

Analysis of anonymised individual-level electronic health and administrative data was carried out on people aged ≥65 years entering a care home between 1 January 2003 and 31 December 2018, provisioned from the Secure Anonymised Information Linkage Databank.

**Results:**

Between 2003 and 2018, 14 493 people with AF aged ≥65 years became new residents in care homes in Wales and 7057 (48.7%) were prescribed OACs (32.7% in 2003 compared with 72.7% in 2018) within 6 months before care home entry. Increasing age and prescription of antiplatelet therapy were associated with lower odds of OAC prescription (adjusted odds ratio [aOR] 0.96 per 1-year age increase, 95% confidence interval [CI] = 0.95 to 0.96 and aOR 0.91, 95% CI = 0.84 to 0.98, respectively). Conversely, prior venous thromboembolism (aOR 4.06, 95% CI = 3.17 to 5.20), advancing frailty (mild: aOR 4.61, 95% CI = 3.95 to 5.38; moderate: aOR 6.69, 95% CI = 5.74 to 7.80; and severe: aOR 8.42, 95% CI = 7.16 to 9.90), and year of care home entry from 2011 onwards (aOR 1.91, 95% CI = 1.76 to 2.06) were associated with higher odds of an OAC prescription.

**Conclusion:**

There has been an increase in OAC prescribing in older people newly admitted to care homes with AF. This study provides an insight into the factors influencing OAC prescribing in this population.

## INTRODUCTION

Atrial fibrillation (AF) disproportionately affects older people, with its prevalence increasing in parallel to population growth and ageing.^1^^,^^2^ Older care home residents represent a growing group of people with AF. Previous estimates for the proportion of older care home residents with a diagnosis of AF have ranged from 7% to 38%.^3^^–^^5^

Atrial fibrillation increases the risk of stroke four-to fivefold;^6^ therefore, stroke prevention is the cornerstone of AF management. This focuses on the prescription of oral anticoagulants (OACs);^7^ however, there is evidence of underprescribing of OACs in care home residents.^3^ The prevalence of anticoagulant use for AF in care homes ranges from 17% to 68% across multiple studies.^3^^,^^5^^,^^8^ With concerns of iatrogenic harm and doubt of the net clinical benefit of treatment, often clinicians face the dilemma of a ‘treatment– risk paradox’ in this vulnerable group. The risk of adverse events is heightened because of the complex interplay between altered pharmacokinetic and pharmacodynamic profiles, frailty, dementia, falls risk, polypharmacy, multimorbidity, and an exponential increase in stroke and bleeding risk with advancing age.^9^^,^^10^ However, the consequences of non-prescription of anticoagulation can be catastrophic; AF-related strokes are often more severe than strokes related to other causes, and people who have an AF-related stroke are more likely to die, experience chronic disability, and require constant nursing care.^11^^–^^13^

**Table:** How this fits in

Available data on factors that influence the decision to prescribe anticoagulation for atrial fibrillation in care home residents is conflicting. This study adds to the body of evidence to suggest that advancing age and concomitant antiplatelet therapy are barriers to anticoagulant prescription in older people newly admitted to care homes. Targeted educational tools on anticoagulant prescribing in older people with atrial fibrillation and an indication for antiplatelet therapy (for example, peripheral vascular disease, ischaemic stroke, and acute coronary syndrome) are needed.

The latest European Society of Cardiology (ESC) guidelines on AF state there is sufficient evidence to support OAC prescribing in older people based on a meta- analysis of landmark trials on non- vitamin K antagonist oral anticoagulants (NOACs) including people aged ≥75 years.^7^^,^^14^^–^^19^ Results from other trials also support the use of warfarin for stroke prevention in people aged ≥75 years with AF.^20^^,^^21^ The ESC guidelines also emphasise that frailty, falls risk, and multimorbidity are not sufficient justification for not prescribing OACs in those who are eligible for treatment.^7^ This study aims to elucidate the factors associated with OAC prescription in older people aged ≥65 years newly admitted to care homes in Wales.

## METHOD

### Study design

This was a retrospective cohort study using anonymised linked data from the Secure Anonymised Information Linkage (SAIL) Databank on CARE home residents with AF (any subtype or atrial flutter) in Wales (SAIL CARE-AF), following the REporting of studies Conducted using Observational Routinely- collected health Data (RECORD) 2015 guidelines (see Supplementary Table S1).^22^

### Data sources

This study utilised anonymised, individual- level population-scale routinely collected electronic health record and administrative data for the population of Wales available within the SAIL Databank.^23^^–^^25^ The data sources included the Welsh Demographic Service Dataset,^26^ the Welsh Longitudinal General Practice (WLGP),^27^ and the Patient Episode Database for Wales (PEDW).^28^ Data were extracted from the PEDW and WLGP using International Classification of Diseases, 10th revision (ICD-10) and Read version 2 codes, respectively (see Supplementary Tables S2 and S3). The WLGP version available to and used by this study contains primary care data with ∼80% coverage of patients and general practices in Wales, and PEDW secondary care data with 100% coverage of patients and services.

### Participants

The (CARE) care home data source within the SAIL Databank is derived from care home information available from the Care Inspectorate Wales (CIW) registry with an assigned Residential Anonymous Linking Field.^29^ This is linked to anonymised address data for individual participants.^30^^–^^32^ The CIW registry 2017/18 was used in this study. Data were extracted for people aged ≥65 years on care home entry with a record of AF (any subtype or atrial flutter) within the PEDW or WLGP (see Supplementary Tables S2 and S3). All participants had a minimum of 12 months data coverage within the WLGP before moving to a care home between 1 January 2003 and 31 December 2018. The cohort was restricted to the first care home entry date to prevent participants being accounted for more than once if they moved in and out of different care homes.

### Covariates

For information on study covariates, see Supplementary Appendix S1.

### Outcomes

The outcome of interest was OAC prescription or non-prescription between 6 months before care home entry and the date of care home entry. This was used as a proxy for prescription/non-prescription at the point of care entry.

### Statistical analyses

Unadjusted logistic regression models were used to explore the association between all covariates and OAC prescription or non-prescription. Unadjusted odds ratios (ORs) were reported with 95% confidence intervals (CIs) and *P*-values. Following the process of purposeful selection, any covariate that was not significant at the level 0.1 and not judged to be a potential confounder by the authors was excluded from the multivariate model.^33^ This threshold was used because conventional significance levels such as 0.05 can fail to identify important variables.^34^ Similar covariates were grouped together and multicollinearity was assessed using the variance inflation factor (see Supplementary Appendix S1). Results were reported as adjusted ORs (aORs) with 95% CIs and *P*-values.

For the covariate major bleeding, a sensitivity analysis was carried out to exclude people that had evidence of OAC prescription and a major bleeding event (defined using ICD-10 codes listed in Supplementary Table S2) within 6 months before care home entry. This attempted to account for any association that may have arisen because of bleeding caused by OACs. All analyses were completed using Stata (version 15).

### Research ethics and information governance

For information on research ethics and information governance see Supplementary Appendix S1.

## RESULTS

### Characteristics of study cohort on care home entry

Between 2003 and 2018, 14 493 people with AF aged ≥65 years who had ≥12 months of primary care data became new residents in care homes in Wales (Table 1 and Supplementary Figure S1). The median age of the cohort was 87.0 years (interquartile range [IQR] 82.6–91.2) and 5103 (35.2%) were male.

**Table 1.** Characteristics of adults with atrial fibrillation aged ≥65 years on care home entry (2003–2018) within the SAIL Databank, by prescription of oral anticoagulation

**Characteristics**	**All participants with AF (N = 14 493)**	**Participants with AF not prescribed OACs (*n* = 7436)**	**Participants with AF prescribed OACs^a^ (*n* = 7057)**	***P*-value**
**Demographics**				
Age, years, median (IQR)	87.0 (82.6–91.2)	87.9 (83.4–92.0)	86.2 (81.9–90.2)	<0.001
Age category, years, *n* (%)				<0.001
65–74	770 (5.3)	341 (4.6)	429 (6.1)	
75–84	4682 (32.3)	2117 (28.5)	2565 (36.3)	
85–94	7859 (54.2)	4157 (55.9)	3702 (52.5)	
≥95	1182 (8.2)	821 (11.0)	361 (5.1)	
Sex, male, *n* (%)	5103 (35.2)	2427 (32.6)	2676 (37.9)	<0.001
WIMD quintile, *n* (%)				1.000
1 (most deprived)	2459 (17.0)	1270 (17.2)	1189 (17.1)	
2	3053 (21.3)	1567 (21.2)	1486 (21.4)	
3	3435 (23.9)	1774 (24.0)	1661 (23.9)	
4	2842 (19.8)	1468 (19.9)	1374 (19.8)	
5 (least deprived)	2554 (17.8)	1316 (17.8)	1238 (17.8)	
Missing	150 (1.0)	41 (0.3)	109 (0.8)	

**Frailty,** ***n* (%)**				<0.001
No frailty	1864 (12.9)	1600 (21.5)	264 (3.7)	
Mild	3714 (25.6)	2142 (28.8)	1572 (22.3)	
Moderate	5145 (35.5)	2329 (31.3)	2816 (39.9)	
Severe	3770 (26.0)	1365 (18.4)	2405 (34.1)	

**Stroke risk, CHA_2_DS_2_-VASc score,^b^ median (IQR)**	4 (3–5)	4 (3–5)	4 (3–5)	<0.001

**Bleeding risk, HAS-BLED score,^b^ median (IQR)**	3 (2–3)	2 (2–3)	3 (2–3)	<0.001

**Social history,** ***n* (%)**				
Smoking history	3996 (27.6)	1620 (21.8)	2376 (33.7)	<0.001
Alcoholism	1223 (8.4)	513 (6.9)	710 (10.1)	<0.001
Heavy drinker	224 (1.5)	118 (1.6)	106 (1.5)	0.680

**Comorbidities^c^**				
Any stroke	2929 (20.2)	1248 (16.8)	1681 (23.8)	<0.001
Stroke (unknown origin)	618 (4.3)	266 (3.6)	352 (5.0)	<0.001
Ischaemic stroke	2232 (15.4)	932 (12.5)	1300 (18.4)	<0.001
Haemorrhagic stroke	336 (2.3)	144 (1.9)	192 (2.7)	0.002
Transient ischaemic attack	796 (5.5)	361 (4.9)	435 (6.2)	<0.001
Myocardial infarction	1131 (7.8)	560 (7.5)	571 (8.1)	0.210
Heart failure	4204 (29.0)	1796 (24.2)	2408 (34.1)	<0.001
Alzheimer’s disease	162 (1.1)	93 (1.3)	69 (1.0)	0.120
Vascular dementia	552 (3.8)	275 (3.7)	277 (3.9)	0.480
Other dementia or unspecified	542 (3.7)	306 (4.1)	236 (3.3)	0.014
Asthma	1214 (8.4)	512 (6.9)	702 (9.9)	<0.001
Chronic obstructive pulmonary disease	1794 (12.4)	811 (10.9)	983 (13.9)	<0.001
Other pulmonary disease	9 (0.1)	<5 (<1)	7 (0.1)	0.081
Peptic ulcer	422 (2.9)	215 (2.9)	207 (2.9)	0.880
Diabetes	728 (5.0)	299 (4.0)	429 (6.1)	<0.001
Renal disease	1052 (7.3)	457 (6.1)	595 (8.4)	<0.001
Liver disease	45 (0.3)	25 (0.3)	20 (0.3)	0.570
Cancer	2236 (15.4)	1055 (14.2)	1181 (16.7)	<0.001
Hypertension	6967 (48.1)	3125 (42.0)	3842 (54.4)	<0.001
Dyslipidaemia	1765 (12.2)	679 (9.1)	1086 (15.4)	<0.001
Peripheral vascular disease	855 (5.9)	352 (4.7)	503 (7.1)	<0.001
Aortic plaque	8 (0.1)	<5 (<1)	5 (0.1)	0.430
Major bleeding	2635 (18.2)	1095 (14.7)	1540 (21.8)	<0.001
Venous thromboembolism	464 (3.2)	93 (1.3)	371 (5.3)	<0.001

a *Prescription within 6 months before care home entry used as a proxy for prescription at the point of care home entry.*

b *CHA_2_DS_2_-VASc, stroke risk assessment scoring one point each for female sex, age 65–74 years, history of heart failure, diabetes, hypertension, vascular disease, and two points each for history of stroke/transient ischaemic attack/venous thromboembolism and age ≥75 years. HAS-BLED, bleeding risk assessment scoring one point each for age >65 years, uncontrolled hypertension, liver disease, renal disease, harmful alcohol use, stroke history, prior major bleeding or a predisposition to bleeding, labile international normalised ratio, and medication usage predisposing to bleeding. See Supplementary Tables S4 and S5 for definitions of the HAS-BLED and CHA_2_DS_2_-VASc risk assessment scores used in this study.*

c *Younger-onset dementia not reported on because of small numbers and risk of resident identification.*

*AF = atrial fibrillation. IQR = interquartile range. OAC = oral anticoagulation. SAIL = Secure Anonymised Information Linkage. WIMD = Welsh Index of Multiple Deprivation.*

Of the total cohort with AF, 7057 (48.7%) had a record of OAC prescription within 6 months before care home entry (Table 1). There were 5734 (81.3%) residents on vitamin K antagonist (VKA), 623 (8.8%) on NOACs, and 700 (9.9%) that switched between VKA or NOAC therapy in the 6 months preceding care home entry (data not shown).

**Figure 1.**
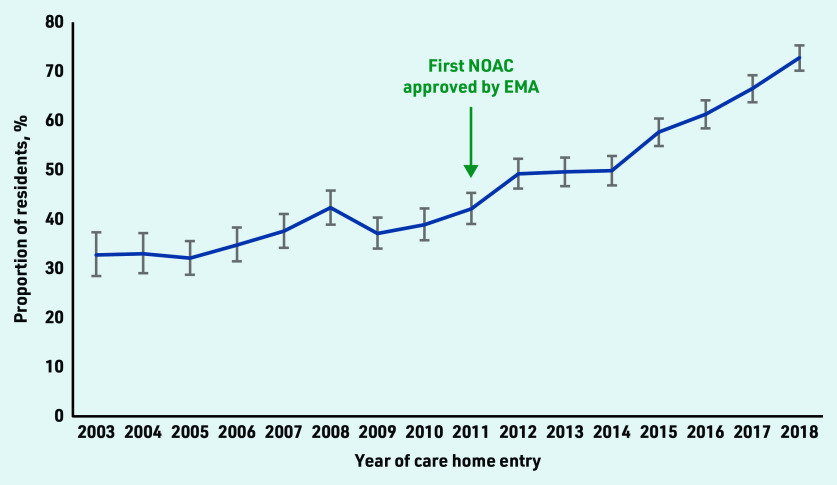
Proportion of care home residents aged *≥*65 years with atrial fibrillation prescribed an oral anticoagulant within 6 months before care home entry between 2003 and 2018. EMA = European Medicines Agency. NOAC = non- vitamin K antagonist oral anticoagulant.

The proportion of residents prescribed OACs increased from 32.7% in 2003 to 72.7% in 2018 (Figure 1). In 2003, all residents were on VKA. In 2018, 385 (33.0%) were on VKA, 228 (19.5%) were on NOACs, and 236 (20.2%) changed between VKA or NOACs before care home entry (data not shown).

Residents prescribed OACs were slightly younger (median age 86.2 years, IQR 81.9– 90.2 versus 87.9 years, IQR 83.4– 92.0) and a higher proportion were male (37.9% versus 32.6%). The median stroke risk (CHA_2_DS_2_-VASc score 4, IQR 3–5) at care home entry was the same in residents prescribed OACs and those residents not prescribed OACs, with a slightly higher median bleeding risk at care home entry for those prescribed OACs (HAS-BLED score 3, IQR 2–3 versus 2, IQR 2–3, respectively) (Table 1).

There was a greater proportion of residents not prescribed OACs on care home entry who were classified as non- frail (21.5% versus 3.7%), whereas more residents categorised with moderate (39.9% versus 31.3%) or severe frailty (34.1% versus 18.4%) were prescribed OACs (Table 1). Severely frail residents more commonly had a history of stroke, transient ischaemic attack (TIA), myocardial infarction, hypertension, heart failure (HF), peripheral vascular disease (PVD), venous thromboembolism (VTE), and diabetes compared with those who were mildly or moderately frail. This translated into a higher median CHA_2_DS_2_- VASc score of 5 (IQR 4–5) for severely frail residents compared with 4 (IQR 3–5) for mild or moderately frail residents (Table 2).

**Table 2.** Advancing frailty and the prevalence of stroke risk factors in care home residents aged ≥65 years with atrial fibrillation

**Category**	**Frailty category on care entry^a^**			

	**No frailty (*n* = 1864)**	**Mild frailty (*n* = 3714)**	**Moderate frailty (*n* = 5145)**	**Severe frailty (*n* = 3770)**
**Stroke risk factors on care entry, *n* (%)^b^**				
Age category, years				
65–74	109 (5.8)	253 (6.8)	242 (4.7)	166 (4.4)
75–84	638 (34.2)	1249 (33.6)	1603 (31.2)	1192 (31.6)
85–94	962 (51.6)	1899 (51.1)	2856 (55.5)	2142 (56.8)
≥95	155 (8.3)	313 (8.4)	444 (8.6)	270 (7.2)
Sex, male	641 (34.4)	1375 (37.0)	1854 (36.0)	1233 (32.7)
Heart failure	297 (15.9)	731 (19.7)	1525 (29.6)	1651 (43.8)
Hypertension	258 (13.8)	1480 (39.8)	2810 (54.6)	2419 (64.2)
Diabetes	46 (2.5)	83 (2.2)	215 (4.2)	384 (10.2)
Myocardial infarction	135 (7.2)	188 (5.1)	380 (7.4)	428 (11.4)
Peripheral vascular disease	36 (1.9)	142 (3.8)	287 (5.6)	390 (10.3)
Venous thromboembolism	38 (2.0)	82 (2.2)	171 (3.3)	173 (4.6)
Transient ischaemic attack	85 (4.6)	165 (4.4)	267 (5.2)	279 (7.4)
Stroke^c^	331 (17.8)	761 (20.5)	1048 (20.4)	789 (20.9)

**Stroke risk assessment on care home entry**				
CHA_2_DS_2_-VASc category,^d ^*n* (%)				
Low moderate	24 (1.3)	42 (1.1)	22 (0.4)	9 (0.2)
Moderate high	1840 (98.7)	3672 (98.9)	5123 (99.6)	3761 (99.8)
CHA_2_DS_2_-VASc score,^d ^ median (IQR)	3 (3–4)	4 (3–5)	4 (3–5)	5 (4–5)

a *Defined using the eFI: no frailty (eFI 0 0.12); mild (eFI >0.12 0.24); moderate (eFI >0.24 0.36); or severe (eFI >0.36) frailty.*

b *Aortic plaque not reported on as a stroke risk factor because of small numbers and risk of resident identification.*

c *Including ischaemic, haemorrhagic stroke, and stroke of unknown origin.*

d *CHA_2_DS_2_-VASc, stroke risk assessment scoring one point each for female sex, age 65–74 years, history of heart failure, diabetes, hypertension, vascular disease, and two points each for history of stroke/transient ischaemic attack/venous thromboembolism and age ≥75 years.*

*eFI = electronic Frailty Index. IQR = interquartile range.*

### Factors associated with OAC prescription

From unadjusted analyses (Table 3), factors associated with OAC non-prescription included increasing age and prescription of antiplatelet therapy. Conversely, stroke risk factors such as prior stroke (ischaemic, haemorrhagic, and stroke of unknown origin), TIA, hypertension, HF, smoking history, VTE, diabetes, and PVD were associated with OAC prescription. Male sex, advancing frailty, harmful alcohol use, major bleeding, cancer, pulmonary disease, renal disease, prescription of non-steroidal anti- inflammatory drugs (NSAIDs), and care home entry from 2011 were also associated with OAC prescription. All variables had a significance level <0.05.

**Table 3.** Association between care home resident characteristics and the prescription of oral anticoagulation for atrial fibrillation^a^

**Characteristics of residents with atrial fibrillation**	**Unadjusted odds ratio (95% CI)**	**Adjusted odds ratio (95% CI)**	***P*-value^b^**
**Demographics**			
Age, per 1-year increase	0.96 (0.96 to 0.97)	0.96 (0.95 to 0.96)	<0.001
Age category			
65–74 years	Reference		
75–84 years	0.96 (0.83 to 1.12)		
85–94 years	0.71 (0.61 to 0.82)		
≥95 years	0.35 (0.29 to 0.42)		
Sex, male	1.26 (1.18 to 1.35)	1.09 (1.01 to 1.18)	0.024
WIMD quintile			
1 (most deprived)	Reference		
2	1.01 (0.91 to 1.13)		
3	1.00 (0.90 to 1.11)		
4	1.00 (0.90 to 1.11)		

**Year of care home entry ≥2011**	2.27 (2.12 to 2.43)	1.91 (1.76 to 2.06)	<0.001

**Frailty**			
No frailty	Reference	Reference	
Mild	4.45 (3.85 to 5.14)	4.61 (3.95 to 5.38)	<0.001
Moderate	7.33 (6.36 to 8.44)	6.69 (5.74 to 7.80)	<0.001
Severe	10.68 (9.23 to 12.36)	8.42 (7.16 to 9.90)	<0.001

**Social history before care home entry**			
Smoking history	1.82 (1.69 to 1.96)	1.16 (1.06 to 1.26)	0.001
Harmful alcohol use^c^	1.51 (1.34 to 1.70)	1.00 (0.88 to 1.14)	0.966

**Medical history before care home entry**			
Dementia^d^	0.91 (0.81 to 1.03)		
Pulmonary disease^e^	1.38 (1.26 to 1.50)	0.94 (0.85 to 1.03)	0.191
Peptic ulcer	1.01 (0.84 to 1.23)		
Cancer	1.22 (1.11 to 1.33)	1.04 (0.94 to 1.15)	0.401
Dyslipidaemia	1.81 (1.63 to 2.00)	1.13 (1.02 to 1.27)	0.025
Haemorrhagic stroke	1.42 (1.14 to 1.76)	1.18 (0.93 to 1.50)	0.183
Ischaemic stroke	1.58 (1.44 to 1.73)	1.51 (1.37 to 1.67)	<0.001
Stroke of unknown origin	1.42 (1.20 to 1.67)	1.32 (1.10 to 1.58)	0.002
Transient ischaemic attack	1.29 (1.12 to 1.49)	1.22 (1.04 to 1.43)	0.012
Myocardial infarction	1.08 (0.96 to 1.22)		
Heart failure	1.63 (1.51 to 1.75)	1.46 (1.35 to 1.58)	<0.001
Diabetes	1.54 (1.33 to 1.80)	1.05 (0.88 to 1.24)	0.603
Renal disease	1.41 (1.24 to 1.60)	0.96 (0.84 to 1.11)	0.582
Liver disease	0.84 (0.47 to 1.52)		
Hypertension	1.65 (1.54 to 1.76)	1.05 (0.98 to 1.13)	0.176
Peripheral vascular disease	1.54 (1.34 to 1.78)	1.09 (0.93 to 1.26)	0.293
Aortic plaque	1.76 (0.42 to 7.35)		
Major bleeding	1.62 (1.48 to 1.76)	1.35 (1.23 to 1.48)	<0.001
Venous thromboembolism	4.38 (3.48 to 5.51)	4.06 (3.17 to 5.20)	<0.001

**Medication history within 6 months before care home entry**			
Prescription of antiplatelet(s) without NSAID(s)	0.88 (0.83 to 0.95)	0.91 (0.84 to 0.98)	0.014
Prescription of NSAID(s) without antiplatelet(s)	2.05 (1.80 to 2.33)	1.75 (1.51 to 2.02)	<0.001

a *Prescription within 6 months before care home entry used as a proxy for prescription at the point of care home entry.*

b *Reported for adjusted odds ratio.*

c *Including alcoholism and heavy drinker.*

d *Including Alzheimer’s disease, vascular dementia, younger-onset dementia, and other or unspecified dementia.*

e *Including asthma, chronic obstructive pulmonary disease, and other pulmonary. NSAID = non-steroidal anti-inflammatory drug. WIMD = Welsh Index of Multiple Deprivation.*

**Figure 2.**
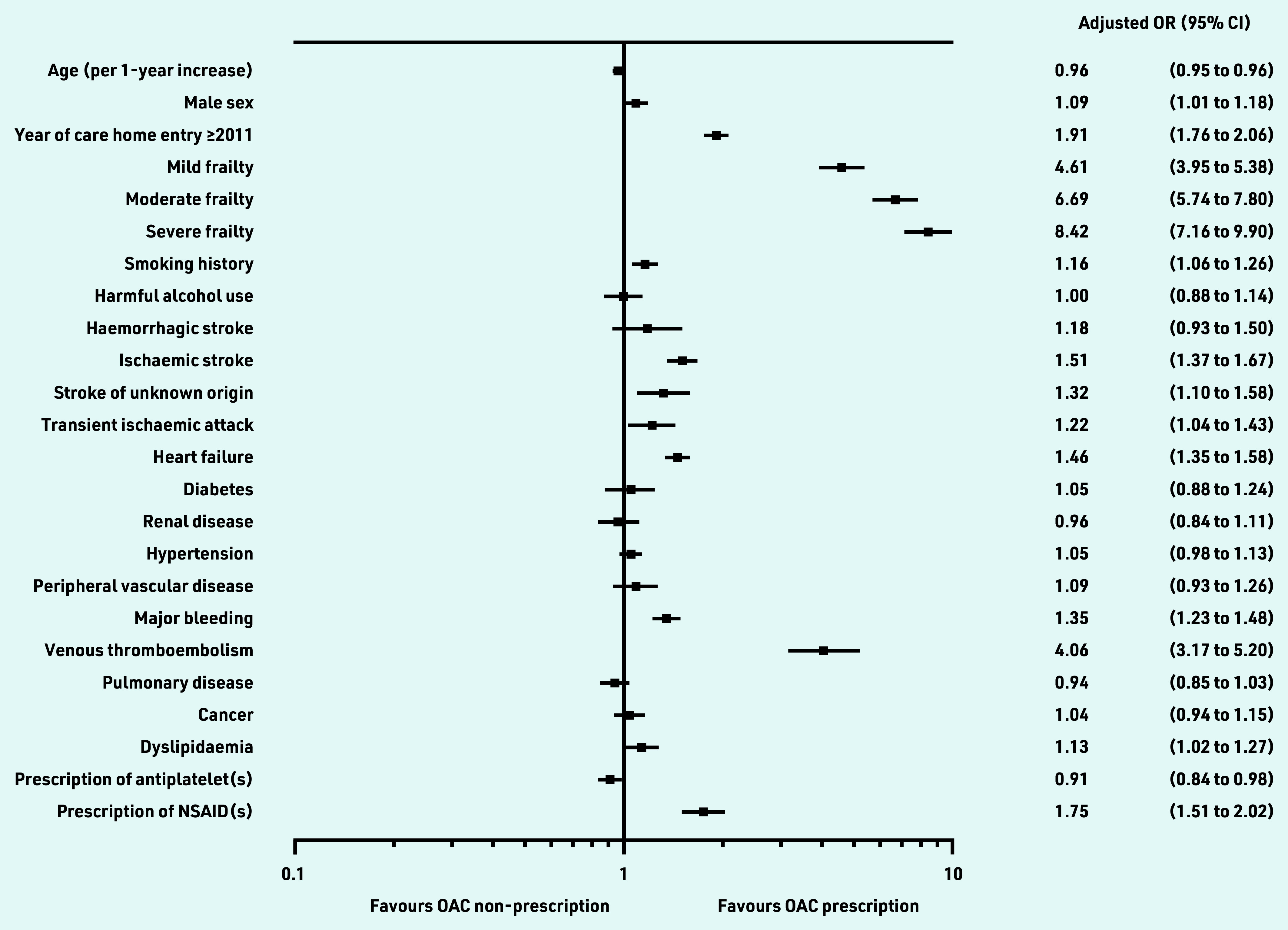
Factors associated with prescription of oral anticoagulation*^a^* in new care home residents aged *≥*65 years with atrial fibrillation, using a multivariable adjusted model. The reference for frailty categories is no frailty. Multivariate model adjusted for dyslipidaemia, smoking history, cancer diagnoses, year of care home entry *≥*2011, and individual components of CHA_2_DS_2_VASc and HAS-BLED risk assessment scores. *^a^* Prescription within 6 months before care home entry used as a proxy for prescription at the point of care home entry. NSAID = non-steroidal anti-inflammatory drug. OAC = oral anticoagulant. OR = odds ratio.

In the multivariate model (Table 3 and Figure 2), advancing age (aOR 0.96 per 1-year increase, 95% CI = 0.95 to 0.96, *P*<0.001) and prescription of antiplatelet therapy remained as factors significantly associated with OAC non-prescription (aOR 0.91, 95% CI = 0.84 to 0.98, *P* = 0.014). Prior VTE (aOR 4.06, 95% CI = 3.17 to 5.20, *P*<0.001), ischaemic stroke (aOR 1.51, 95% CI = 1.37 to 1.67, *P*<0.001) and HF (aOR 1.46, 95% CI = 1.35 to 1.58, *P*<0.001) were the stroke risk factors most strongly associated with OAC prescription. Dyslipidaemia, smoking history, stroke of unknown origin, and TIA were also other stroke risk factors independently associated with OAC prescription. There was no significant association found between hypertension and OAC prescription.

Other independent predictors of OAC prescription included male sex (aOR 1.09, 95% CI = 1.01 to 1.18, *P* = 0.024), advancing frailty (mild: aOR 4.61, 95% CI = 3.95 to 5.38, *P*<0.001; moderate: aOR 6.69, 95% CI = 5.74 to 7.80, *P*<0.001; severe: aOR 8.42, 95% CI = 7.16 to 9.90, *P*<0.001), major bleeding (aOR 1.35, 95% CI = 1.23 to 1.48, *P*<0.001), prescription of NSAIDs (aOR 1.75, 95% CI = 1.51 to 2.02, *P*<0.001) and care home entry from 2011 onwards (aOR 1.91, 95% CI = 1.76 to 2.06, *P*<0.001).

The variance inflation factor was <1.5 for all covariates (see Supplementary Table S6). When age was included as a categorical variable, the same covariates were identified as predictors of OAC prescription or non-prescription (see Supplementary Table S7). When people who had a major bleeding event and evidence of OAC prescription within 6 months before care home entry were excluded, the positive association between OAC prescription and major bleeding was attenuated (aOR 1.12, 95% CI = 1.01 to 1.23, *P* = 0.028) (see Supplementary Figure S2).

## DISCUSSION

### Summary

This study found that between 2003 and 2018, less than half of new care home residents with AF aged ≥65 years were prescribed OACs. The proportion of new residents prescribed an OAC within 6 months before care home entry more than doubled from 2003 to 2018. Increasing age and prescription of antiplatelet therapy were independently associated with OAC non- prescription. In contrast, advancing frailty, prior VTE, and year of care home entry after 2011 were the strongest predictors independently associated with OAC prescription.

### Strengths and limitations

To the authors’ knowledge, this is one of the largest population studies conducted exclusively in new care home residents that aims to elucidate the factors associated with OAC prescription for AF. Study limitations pertain to the observational design and use of routinely collected data. Data can only give insight into association rather than causation, and the direction of causality cannot be conferred. It is possible that some diagnoses were missed using Read and ICD-10 codes, or classified incorrectly. In the present study it is anticipated there may be a greater proportion of residents with dementia than actually reported, and the number of people with uncontrolled hypertension may have been overestimated using the study’s definition. Investigation of the temporal association between major bleeding and OAC prescription was limited because it was not possible to confirm whether OACs were prescribed before or after the major bleeding event, or what time elapsed between the two. It was not possible to explore temporal associations between NSAID prescription or harmful alcohol use and OAC prescription as these data were not available in SAIL.

### Comparison with existing literature

When comparing the findings in the present study to other care home studies, some of the results are conflicting. One systematic review^3^ of observational studies in care homes found the majority of studies reported older age,^35^^–^^40^ falls/fall risk,^39^^,^^40^ and dementia/cognitive impairment^36^^–^^39^^,^^41^ as independent predictors of anticoagulant non-prescription, but a number of studies did not.^37^^,^^40^^–^^42^ Studies also reported previous stroke/TIA^35^^,^^36^^,^^38^^,^^39^^,^^43^ and VTE^35^^,^^36^^,^^39^ as independent predictors of anticoagulant prescription, but again, this was not found in all studies.^42^ Two studies reported no association between anticoagulant prescription and antiplatelet therapy in multivariate analysis,^36^^,^^42^ but one study reported antiplatelet therapy as an independent predictor of anticoagulant non-prescription.^35^ Inconsistencies in results likely relate to differences in study methods to establish residents’ medical and medication history. Another explanation is diversity across care home settings, where resident characteristics, clinical practice, and perception of OAC use will differ.

Over the past decade, guidance on stroke prevention management for AF has changed. NOACs became available in Europe in 2011, and are now recommended in preference to VKA therapy.^7^ Devoid of complex monitoring requirements, NOACs have improved accessibility to OAC therapy and this is reflected in the study results; people who entered a care home in the post-NOAC era (from 2011 onwards) were significantly more likely to be prescribed OAC therapy. The standpoint on concomitant prescription of antiplatelet and OAC therapy has remained unchanged, with guidelines advising against this to minimise the risk of bleeding.^7^^,^^44^ An exception to this is in the event of acute coronary syndrome or percutaneous coronary intervention where antiplatelet therapy is indicated alongside OACs for up to 12 months post-event.^7^^,^^44^ Caution should also be applied when prescribing NSAIDs alongside OAC therapy because of the increased risk of bleeding.^7^ Although the results of this study suggest an aversion to concomitant prescription of OAC and antiplatelet therapy, this does not appear to be the case for NSAID therapy. NSAIDs are usually prescribed for a short duration to manage acute pain arising from inflammatory conditions. Without being able to distinguish residents prescribed an acute course of NSAID from those regularly prescribed NSAIDs alongside their OAC therapy, the findings must be interpreted cautiously. Concomitant prescription is not an absolute contraindication, so it is possible the results reflect an acceptance to prescribe short courses of NSAIDs alongside OAC therapy in some individuals.

The study finding that each 1-year age increase is associated with a 4% reduction in OAC prescription verifies ongoing concerns about underprescribing in older people because of misperceptions of the risk of adverse effects.^45^ Ageing is a prominent non-modifiable risk factor for stroke,^6^^,^^7^ and any reduction in OAC prescription as a result of older age will have clinical consequences. Recently, a large registry study verified OAC safety in people aged >90 years with a history of chronic kidney disease and intracranial haemorrhage.^46^

There is considerable overlap between bleeding and stroke risk factors, and people with AF and a history of bleeding or intracranial haemorrhage remain at a high ischaemic stroke risk.^7^ This may explain the positive association found between major bleeding and OAC prescription. Although this study found an expected rise in OAC prescribing for increasingly frail people, further work is needed to investigate the interaction with deprivation and other socioeconomic and demographic factors to assess potential inequalities in prescribing across these groups. Nevertheless, this finding provides an interesting insight on prescribing patterns before definitive guidance on OAC prescription in people with frailty was detailed by the ESC in 2020, which states that frailty should not be a barrier to OAC prescription.^7^ Another electronic health record study of 58 204 people with AF aged ≥65 years in England also reported a positive association between advancing electronic Frailty Index frailty category and OAC prescription (mild: aOR 1.84, 95% CI = 1.72 to 1.96; moderate: aOR 2.34, 95% CI = 1.18 to 2.50; and severe: aOR 2.51, 95% CI = 2.33 to 2.71).^47^ One explanation may be that frailer adults are more frequently reviewed by clinicians, so there are less likely to be omissions in OAC prescribing.

### Implications for research and practice

This study provides an important insight into the factors that influence OAC prescribing for new care home residents with AF, a high-risk group that is underrepresented in research. The proportion of new residents prescribed OAC therapy has increased with the introduction of NOACs, but OAC prescription rates are still suboptimal. There is a need for future research to elucidate other barriers to OAC prescription, and further explore temporal associations between OAC prescription and falls, alcohol use, and prescription of antiplatelet/NSAID therapy.

## Data

The data used in this study are available in the SAIL Databank at Swansea University, Swansea, UK. Analytic code could be provided for future work after agreed acknowledgement in any future publications. All proposals to use SAIL data are subject to review by an IGRP. Before any data can be accessed, approval must be given by the IGRP. The IGRP ensures appropriate use of SAIL data. When access has been approved, it is gained through a privacy-protecting safe haven and remote access system referred to as the SAIL Gateway. An application process should be followed by anyone who would like to access data via SAIL at: https://saildatabank.com/data/apply-to-work-with-the-data.
